# Interaction of calcium binding protein S100A16 with myosin-9 promotes cytoskeleton reorganization in renal tubulointerstitial fibrosis

**DOI:** 10.1038/s41419-020-2337-z

**Published:** 2020-02-24

**Authors:** Hui Sun, Anran Zhao, Min Li, Hao Dong, Yifei Sun, Xue Zhang, Qian Zhu, Ashfaq-Ahmad-Shah Bukhari, Changchun Cao, Dongming Su, Yun Liu, Xiubin Liang

**Affiliations:** 1 Department of Pathophysiology, Nanjing Medical University, 211166 Nanjing, China; 2 Departments of Pathology, The Affiliated Hospital of Nantong University, 226001 Nantong, China; 3 Department of Pathology, Nanjing Medical University, 211166 Nanjing, China; 4 Department of Nephrology, The Affiliated Sir Run Run Hospital of Nanjing Medical University, 211166 Nanjing, China; 5 Center of Pathology and Clinical Laboratory, The Affiliated Sir Run Run Hospital of Nanjing Medical University, 211166 Nanjing, China; 6 Department of Geratology, The First Affiliated Hospital of Nanjing Medical University, 210029 Nanjing, China

**Keywords:** Mechanisms of disease, Chronic kidney disease

## Abstract

Renal fibrosis arises by the generation of matrix-producing fibroblasts and myofibroblasts through the epithelial–mesenchymal transition (EMT), a process in which epithelial cells undergo a transition into a fibroblast phenotype. A key feature of the EMT is the reorganization of the cytoskeletons, which may involve the Ca^2+^-binding protein S100A16, a newly reported member of the S100 protein family. However, very few studies have examined the role of S100A16 in renal tubulointerstitial fibrosis. In this study, S100A16 expression was examined by immunohistochemical staining of kidney biopsy specimens from patients with various nephropathies and kidney tissues from a unilateral ureteral obstruction (UUO) mouse model. Renal histological changes were investigated in S100A16^Tg^, S100A16^+/−^, and WT mouse kidneys after UUO. The expression of epithelia marker E-cadherin, mesenchymal markers N-cadherin, and vimentin, extracellular matrix protein, and S100A16, as well as the organization of F-actin, were investigated in S100A16 overexpression or knockdown HK-2 cells. Mass spectrometry was employed to screen for S100A16 binding proteins in HK-2 cells. The results indicated that S100A16 is high expressed and associated with renal tubulointerstitial fibrosis in patient kidney biopsies and in those from UUO mice. S100A16 promotes renal interstitial fibrosis in UUO mice. S100A16 expression responded to increasing Ca^2+^ and interacted with myosin-9 during kidney injury or TGF-β stimulation to promote cytoskeleton reorganization and EMT progression in renal tubulointerstitial fibrosis. Therefore, S100A16 is a critical regulator of renal tubulointerstitial fibroblast activation and is therefore a potential therapeutic target for the treatment of renal fibrosis.

## Introduction

Renal fibrosis, particularly tubulointerstitial fibrosis, is the common final outcome of almost all progressive chronic kidney diseases. Kidney interstitial fibrosis plays a determining role in the development and progression of kidney injury^1,2^. The process of renal fibrosis depends on the pathophysiological changes associated with the epithelial–mesenchymal transition (EMT)^3^. This transition of epithelial cells into mesenchymal cells occurs in a series of multiple steps that include dissolution of epithelial cell–cell tight adhesions, reorganization of the actin cytoskeleton, increases in cell matrix contacts, and enhancement of migration capabilities. Epithelial cells that are induced by injury factors or cytokines to undergo the EMT lose a specific cell marker (E-cadherin) and acquire the characteristics of mesenchymal cells or myofibroblasts (indicated by the markers N-cadherin, vimentin, and α-SMA)^4^. Increasing evidence now indicates that the actin cytoskeleton undergoes a dynamic reorganization to provide the necessary cell structural changes and mechanical strength required for epithelial cells with an apical-basal polarity to transition into spindle-like mesenchymal phenotypes during the EMT process^5,6^. However, the regulatory mechanisms are not completely understood.

The S100 family proteins, consisting of at least 21 members, have been reported to be associated with a variety of human diseases, including cardiomyopathies, neurodegenerative diseases, inflammation disorders and cancers^7,8^. These proteins S100A16, a newly discovered member of the S100 family, is widely expressed in various human tissues and organs^9,10^, and its overexpression has been linked to increased adipocyte proliferation and lipogenesis^11,12^. S100A16 also appears to participate in glucose metabolism disorders^9,13^, but its full physiological and pathogenic function remains unclear. S100 proteins including S100A16 contain Ca^2+^-binding EF-hand motifs (helix-loop-helix structural domain) to interact with calcium for its biological function^14^. The calcium signaling involved in cytoskeleton reorganization was reported in many previously studies^15–17^. However, its ability to interact with cytoskeletal proteins suggests that S100A16 may play a role in cell type transition through regulation of the cytoskeleton.

In this study, we investigated the potential roles of S100A16 in renal fibrosis. We hypothesized that S100A16 regulate the EMT process of renal tubular cells. Patient kidney biopsies, UUO mice model and HK-2 cells were used in the study. Our results indicated that S100A16 was upregulated in all human patient kidney samples, and that S100A16 responded to Ca^2+^ increases and interacted with myosin-9 during kidney injury or following TGF-β stimulation. S100A16 promoted cytoskeleton reorganization and participated in the EMT progression in renal tubulointerstitial fibrosis.

## Materials and methods

### Reagents, plasmid constructs, and antibodies

Antibodies against S100A16, myosin-9, E-cadherin, N-cadherin, vimentin, collagen I, and GAPDH were purchased from Proteintech (Chicago, USA). Antibodies against fibronectin and α-SMA were purchased from Sigma-Aldrich (St Louis, MO). Other antibodies, including normal rabbit IgG for immunoprecipitation and secondary mouse or rabbit antibodies for western blotting, were purchased from Thermo Fisher (Waltham, UK). TGF-β was purchased from R&D (Minneapolis, USA). Rhod-2 AM and BAPTA-AM were purchased from Sigma-Aldrich (St Louis, MO). The constructs of shRNA-S100A16 and shRNA-scrambled were designed and cloned into pcDNA6.2. All plasmid constructs were confirmed by DNA sequencing.

### Mice and animal models

Male C57BL/6J (WT) mice, heterozygous S100A16 knockout (S100A16^+/−^) mice, and S100A16 transgenic (S100A16^Tg^) mice (*n* = 16 each group) weighing approximately 20–24 g were acquired from the Animal Center of Nanjing Medical University. All protocols for animal experimentation and maintenance adhered to the guidelines of the Institutional Animal Care and Use Committee at Nanjing Medical University^12,13,18^. S100A16^Tg^ mice were engineered to overexpress S100A16 under the control of the PCAG promoter. Mice at 12 weeks of age received left ureteral ligation with 6-gauge silk sutures. The mice were euthanized at days 7 after unilateral ureteral obstruction (UUO), and the UUO kidneys, as well as the contralateral kidneys, were removed. The serum creatinine (Scr) and blood urea nitrogen (BUN) levels of all animals were determined with an automated biochemical analyzer (7600-DDP-ISE; Hitachi Software Engineering, Yokohama, Japan). One portion of the kidney was fixed in 10% formalin and embedded in paraffin for histologic and immunohistochemical staining. Another portion was snap-frozen in liquid nitrogen and stored at −80 °C for extraction of RNA and protein.

### Cell culture and treatment

Renal tubular epithelial cells (HK-2 cells) were cultured in DMEM-F12 medium (Gibco America) supplemented with 10% fetal bovine serum (FBS) (BI, Israel). The cells were ready for treatment when the cell confluence was 60–70% in complete medium containing 10% FBS. For cell transfection, the constructed vectors were transfected into HK-2 cells using Lipo 3000 (Roche) followed instruction. Gene expression or knockdown was examined by western blotting at 24–48 h after cell transfection.

### Stable cell line

For the generation of the stable cell line of S100A16 overexpression, the lentivirus of S100A16 was mixed with transfection reagent and added to the HK-2 cells. The cells were transfected for 48 h and then cultured in purinomycin-containing culture medium for 14 days to obtain an HK-2 cell line with stable overexpression of S100A16.

### Western blotting and co-IP assay

The proteins in lysates from mice or HK-2 cells were resolved by 8 or 12% SDS-PAGE on gels containing 50 mM Tris-HCl, pH 6.8 or pH 8.8, 10% ammonium persulfate 30% bis-acrylamide, 10% SDS and 1% TEMED. Unbound sites were blocked with 5% nonfat milk in Tris-buffered saline (TBS) with 0.1% Tween-20 for 1.5 h at room temperature. The blotted membrane was probed with the appropriate primary antibodies at various dilutions, and then incubated with horseradish peroxidase (HRP)-conjugated secondary antibodies. The immunorecognition signals were detected using enhanced chemiluminescence (ECL) and were acquired using an Image Quant ECL system (PerkinElmer Life Sciences, Wellesley, MA). Western blotting data were quantified with Image Lab software. For immunoprecipitation (IP) studies, lysates were collected from wild type or transiently transfected HK-2 cells and lysed with NP40 lysis buffer containing 10 mM Tris-HCl (pH 7.4) and 10 mM NaCl. The protein concentrations of the lysates were determined by bicinchoninic acid assays, and then 0.5 mg protein was incubated with specific antibodies of target gene overnight at 4 °C. The antibody/protein mixture was then incubated with 100 μl protein A beads for 2–6 h. The resulting immunocomplexes were washed with NP40 and degenerated with 2× SDS loading buffer containing 250 mM Tris-HCl pH 6.8, 10% SDS, 50% glycerin, 0.5% bromophenol blue, and 5% β-mercaptoethanol, and then subjected to immunoblotting.

### RNA extraction, purification, and real-time PCR analyses

Total RNA was extracted from cells using Trizol reagent in accordance with the manufacturer’s instruction (Thermo, USA). RNA (1 µg) was then reversely transcribed to cDNA with ReverTra Ace@ qPCR RT kit (TOYOBO, China). PCR was performed using SYBR Green Master Mix (Applied Biosystems) and the Applied Biosystems StepOne Plus Real-Time PCR system. Gene expression was normalized to housekeeping gene GAPDH, and fold change in expression relative to the control group was calculated using the 2^−ΔΔCt^ method. The following primer sequences were used: human S100A16: forward 5′-TTG GAT CCG GAG ATG TCA GAC TGC TAC AC-3′ and reverse 5′-TTA CGC GTA AAG GGG TCT CTA GCT GCT G-3′; human GAPDH: forward 5′-ATG GGG AAG GTG AAG GTC G-3′ and reverse 5′-GGG GTC ATT GAT GGC AAC AAT A-3′.

### Histology and immunohistochemistry

Human kidney specimens were acquired from diagnostic renal biopsies collected during operations conducted in the SIR RUN RUN Hospital of Nanjing Medical University. Normal kidney tissue from patients with renal carcinoma who underwent nephrectomy were used as a comparison. Mouse kidney samples were fixed and embedded in paraffin and sectioned at 3 or 4 μM thickness for hematoxylin and eosin (HE) and Masson staining. Immunohistochemical staining was performed using routine protocols using rabbit polyclonal anti-S100A16 antibody. After incubation with the primary antibody at 4 °C overnight, the slides were then stained with horseradish peroxidase–conjugated secondary antibody.

### Immunofluorescence staining

For immunofluorescence staining, HK-2 cells were fixed in 4% paraformaldehyde for 60 min at room temperature, followed by three extensive washes with PBS to remove any debris. The cells were then blocked with 1% BSA for 1.5 h at 37 °C and incubated overnight at 4 °C with anti-S100A16 antibody and mouse anti-Myh9 antibody. After three washes with PBS, the cells were incubated with appropriate secondary antibodies for 30 min at 37 °C. The cells were then stained with 4′,6-diamidino-2-phenylindole for 2 min and washed with PBS. All images were obtained using an Olympus confocal microscope and processed using Photoshop software.

### Mass spectrometry

HK-2 cells stably overexpressing S100A16 or transfected with scrambled oligonucleotide were lysed with RIPA and the cell lysates were immunoprecipitated with anti-S100A16 antibody. Protein digestion, labeling, mass spectrometry data acquisition, and identification were completed in the analysis center of Nanjing Medical University. Briefly, a LTQ-Orbitrap instrument (Thermo Fisher, USA) connected to a Nano ACQUITY UPLC system was used to analyze the labeled the peptide samples, as described previously, as well as the acquired MS/MS spectra and parameters^19,20^.

### Antibody transfection

A Pro-Ject™ Protein Transfection Reagent Kit (Thermo Scientific) was used to deliver the MYH9 antibody into the HK-2 cells, following the manufacturer’s instructions. In brief, 5 µg of anti-MYH9 antibody was diluted in PBS buffer (20 mM sodium phosphate, 150 mM NaCl, pH 7.4) and hydrated with prepared Pro-Ject™ Reagent at room temperature for 5 min. After addition of 1 ml of serum-free medium to the Pro-Ject™-Reagent–protein complex, the mixture was added to HK-2 cells in a well of a six-well plate.

### Ca^2+^ fluorescent probe loading assay

Changes in intracellular calcium concentration [Ca^2+^] were measured by seeding transfected HK-2 cells into a six-well plate, adding TGF-β (20 ng/ml) or BAPTA-AM (10 µM) to the medium, and culturing for 36 h. The growth medium was removed and cells were washed three times in PBS buffer without sans calcium and magnesium. Then the cells were cultured for 30 min in the Rhod-2 AM dye buffer, a rhodamine-based Ca^2+^ indicator probe, and then kept in a dye-free buffer for 30 min. Fluorescence intensity was determined using a fluorescence microplate reader. All cell numbers are adjusted by DAPI. Fluorescence images were obtained using an Olympus fluorescence microscope and processed using Photoshop software.

### Statistical analysis

GraphPad Prism 6.0 (GraphPad Software, Inc. La Jolla, CA, USA) was used for statistical analyses. All differences were considered statistically significant if *p* < 0.05, and the data were presented as mean ± standard deviation. Data were statistically analyzed using one-way ANOVA with a Bonferroni correction, followed by Fisher’s exact test for comparison of two groups.

## Results

### Increased S100A16 expression occurs in multiple types of clinical nephropathy and in a mouse UUO model

**Fig. 1:**
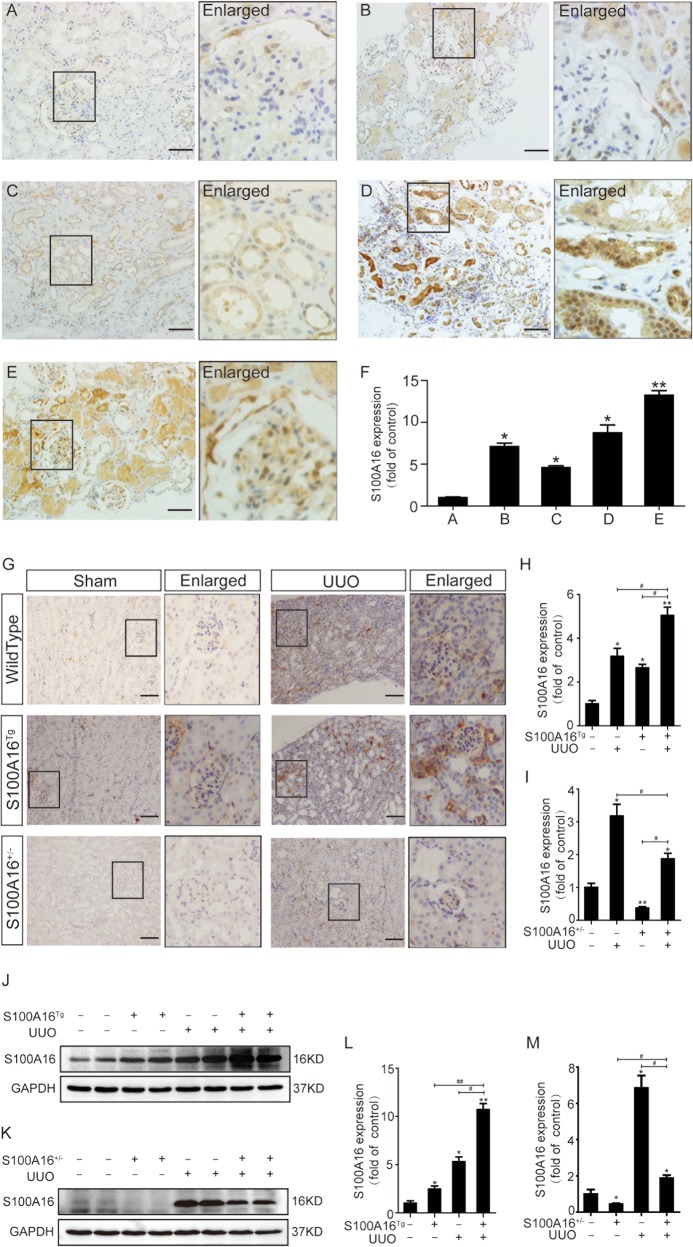
Increased S100A16 expression in multiple types of clinical nephropathy and in a mouse UUO model. **a**–**e** Immunohistochemical staining of S100A16 in kidney biopsy specimens from patients with kidney disease. Scale bar = 50 μm. **f** Semi-quantitative analysis of S100A16 protein expression levels. *p < 0.05, **p < 0.01 com*p*ared with normal human kidneys. **g** Representative images for immunohistochemical staining of S100A16 in the obstructed kidneys of wild type mice, S100A16^Tg^ mice, and S100A16^+/−^ mice. Scale bar = 50 μm. **h**, **i** Semi-quantitative analysis of S100A16 protein expression in S100A16^Tg^ and S100A16^+/−^ UUO kidneys compared with wild type sham groups. *p < 0.05, **p < 0.01 compared with wild type sham groups; ^#^*p* < 0.05. **j**, **k** Representative bands of western blots showing S100A16 protein abundance in obstructed transgenic murine kidneys. **l**, **m** Semi-quantitative analysis of S100A16 protein expression levels. *p < 0.05, **p < 0.01 compared with a wild type sham groups; ^#^*p* < 0.05, ^##^*p* < 0.01.

The clinical relevance of S100A16 in renal disease was investigated by immunohistochemical staining of kidney biopsy specimens from patients with various nephropathies, including mild mesangial proliferative glomerulonephritis, mesangial proliferative glomerulonephritis (MsPGN), focal segmental glomerulosclerosis (FSGS), ureteral calculi with chronic glomerulonephritis, and diabetic nephropathy IV stage, and compared with those from normal human kidneys. As shown in Fig. 1a–e, the S100A16 protein was weak in normal human kidneys biopsies (Fig. 1a); whereas the strong S100A16 signals was detected in all specimens from clinical nephropathy samples (Fig. 1b–e). Notably, S100A16 expression predominated in the renal tubular epithelium in diseased human kidneys. The quantified data for the expression of S100A16 are shown in Fig. 1f.

The pathogenic relevance of S100A16 in renal disease was further assessed by immunostaining of kidney samples from UUO mice, a commonly used animal model of interstitial fibrosis. As illustrated in supplemental Table 1, BUN levels in WT, S100A16^Tg^ and S100A16^+/−^ mice were significantly elevated after UUO compared to before UUO. Scr levels in WT and S100A16^Tg^ mice were significantly increased after UUO, but Scr level in S100A16^+/−^ didn’t show significantly change after UUO. The expression of S100A16 become detectable in UUO kidneys 7 days post induction (Fig. 1g). An additional upregulation of S100A16 expression was observed in the kidneys of both S100A16^Tg^ and S100A16^+/−^ mice after UUO compared with S100A16 expression before UUO. The quantified data for S100A16 expression in S100A16^Tg^ and S100A16^+/−^ UUO kidneys are shown in Fig. 1h, i. Western blotting using of lysates from S100A16^Tg^ UUO kidneys, S100A16^+/−^ UUO kidneys, or WT kidneys and compared with a sham group (Fig. 1j–m) confirmed the immunostaining findings. These results suggested that S100A16 expression is induced in injured kidneys.

### S100A16 promotes renal tubulointerstitial fibrosis in UUO mice

**Fig. 2:**
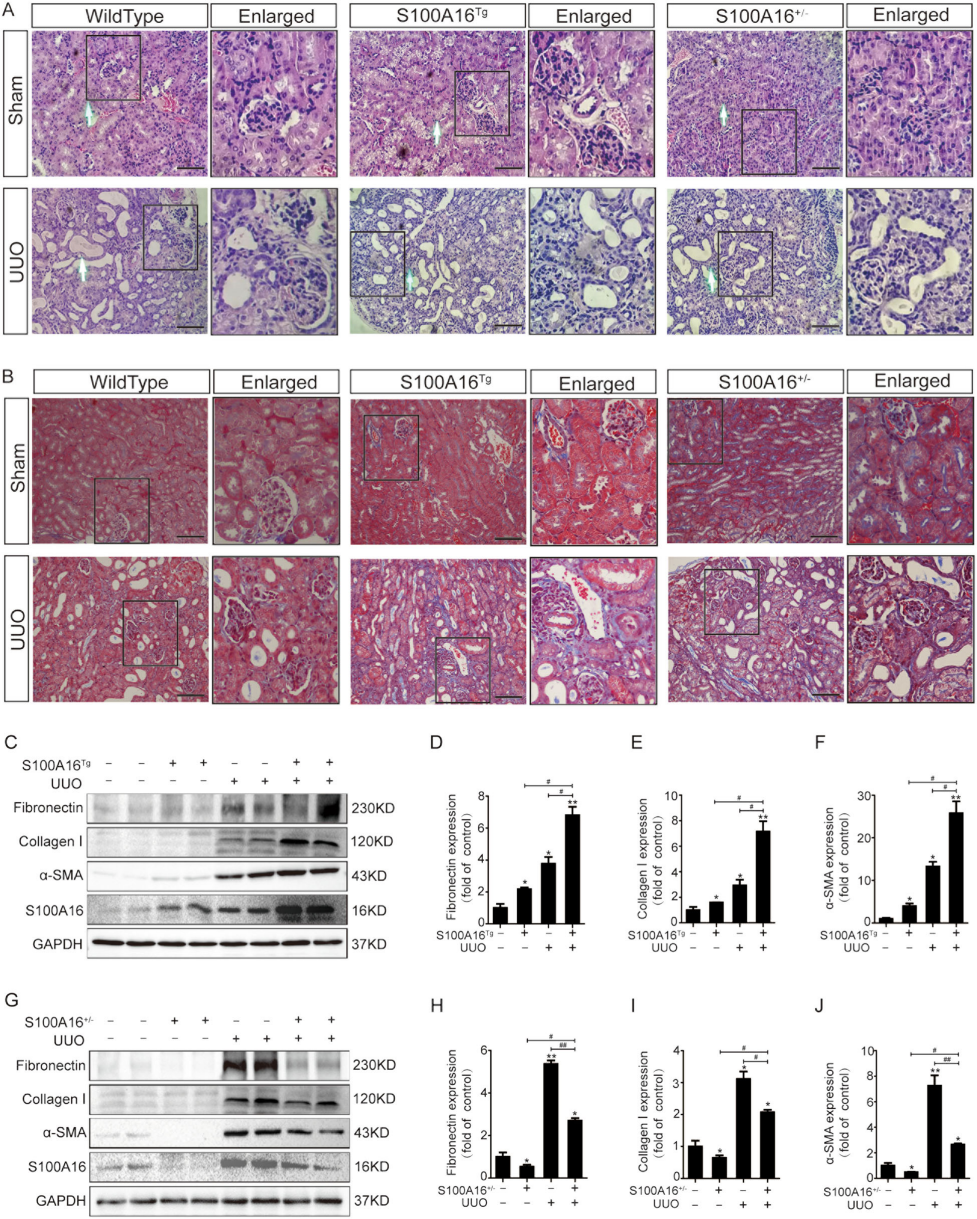
S100A16 promotes renal interstitial fibrosis in UUO mice. **a**, **b** Representative micrographs of hematoxylin and eosin (HE) and Masson’s trichrome stained kidney tissues demonstrate renal injury in S100A16^Tg^, S100A16^+/−^, and wild type mouse kidneys. Scale bar = 50 μm. **c**–**f** Representative bands (two cases) of western blots showing the expression of fibronectin, collagen I, and α-SMA in the obstructed kidneys of wild type or S100A16^Tg^ mice. *p < 0.05, **p < 0.01 vs. wild type sham groups; ^#^*p* < 0.05. **g**–**j** Representative bands (two cases) of western blots showing the expression of fibronectin, collagen I, and α-SMA in the obstructed kidneys of wild type or S100A16^+/−^ mice. *p < 0.05, **p < 0.01 vs. wild type sham groups; ^#^*p* < 0.05, ^##^*p* < 0.01.

We next investigated if A100A16 participates in renal interstitial fibrosis by using S100A16^Tg^, S100A16^+/−^, and WT mice for UUO. The renal tissues of the WT UUO mice showed positive interstitial inflammation and fibrosis when stained with HE (Fig. 2a) and Masson’s trichrome (Fig. 2b). The S100A16^Tg^ mice exhibited a more severe pathological injury than the WT; whereas the S100A16^+/−^ mice showed a marked reduction in inflammatory cell infiltration and interstitial fibrosis. Consistently, western blot of kidney tissues showed that overexpression of S100A16 in Tg mice promoted the expression of renal fibrosis-related proteins including fibronectin, α-SMA, and collagen I proteins (Fig. 2c–f), in contrast, S100A16 knockout significantly attenuated their expressions (Fig. 2g–j).

### S100A16 accentuates the fibrogenic effect of TGF-β in tubular epithelial cells by promoting the epithelial-mesenchymal transition

**Fig. 3:**
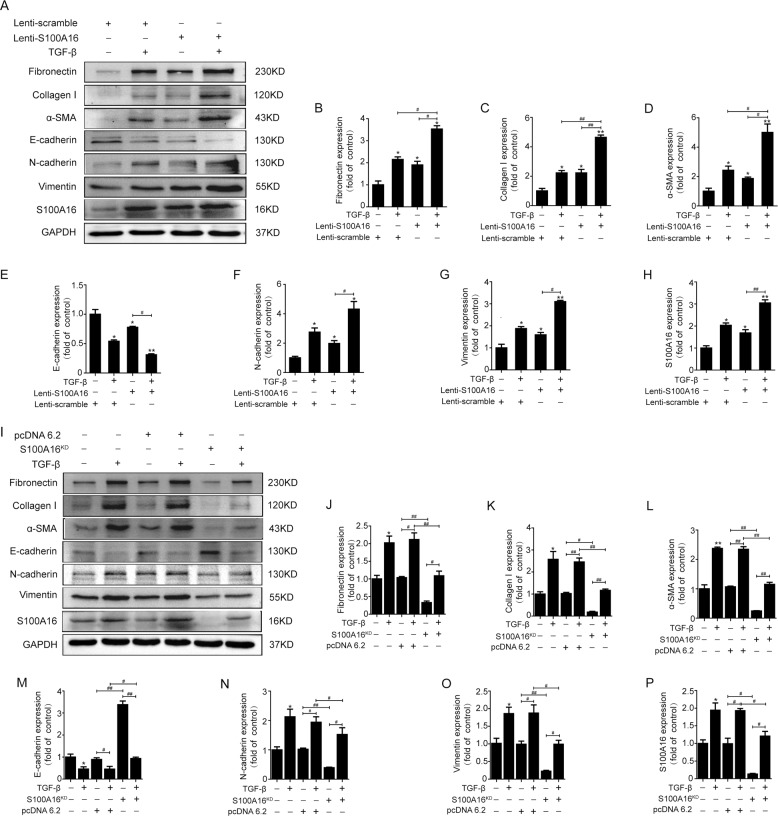
S100A16 accentuates the TGF-β-induced EMT and fibrogenic effects in HK-2 cells. **a**–**h** Representative bands of western blots showing the expression of fibronectin, collagen I, α-SMA, E-cadherin, N-cadherin, vimentin, and S100A16 in lenti-scrambled and lenti-S100A16 virus-treated HK-2 cells. *p < 0.05, **p < 0.01 vs. scrambled; ^#^*p* < 0.05, ^##^*p* < 0.01. **i**–**p**^.^ Representative bands of western blots showing the expression of fibronectin, collagen I, α-SMA, E-cadherin, N-cadherin, vimentin, and S100A16 in normal, vector-transfected, and S100A16^KD^ HK-2 cells. *p < 0.05, **p < 0.01 vs. normal controls; ^#^*p* < 0.05, ^##^*p* < 0.01.

To gain more molecular insights on S100A16′s roles in kidney injury, we examined the expressions of genes involved in the epithelial-mesenchymal transition process using HK-2 cells overexpressing S100A16. The western blotting results (Fig. 3a) reveal a marked induction of fibronectin, α-SMA, and collagen I by TGF-β in lenti-scrambled HK-2 cells and similar results in HK-2 cells expressing lenti-S100A16. S100A16 overexpression dramatically promoted the fibrogenic actions of TGF-β by driving an additional upregulation of fibronectin, α-SMA, and collagen I. The quantified data for fibronectin, collagen I, and α-SMA expression are shown in Fig. 3b–d. Of note, S100A16 overexpression decreased E-cadherin expression while inducing N-cadherin and vimentin expression in HK-2 cells, suggesting that the cells had undergone a phenotypic change due to the EMT (Fig. 3a, e–h). Knockdown of S100A16 expression in HK-2 cells by transfection with S100A16 shRNA, on the other hand, markedly attenuated the TGF-β-induced fibrosis-related gene expression, and significantly increased E-cadherin expression and decreased N-cadherin and vimentin expression (Fig. 3i–p). The efficacy of overexpress and knockdown S100A16 was evaluated by real-time PCR (Supplemental Fig. 1). These results hinted that S100A16 may participate in the TGF-β-induced fibrogenic process associated with the EMT in tubular epithelial cells.

### S100A16 promotes the EMT in the fibrotic kidney

We next looked at EMT markers including E-cadherin, N-cadherin, and vimentin in UUO kidneys in S100A16^Tg^ and S100A16^+/−^ mice.

**Fig. 4:**
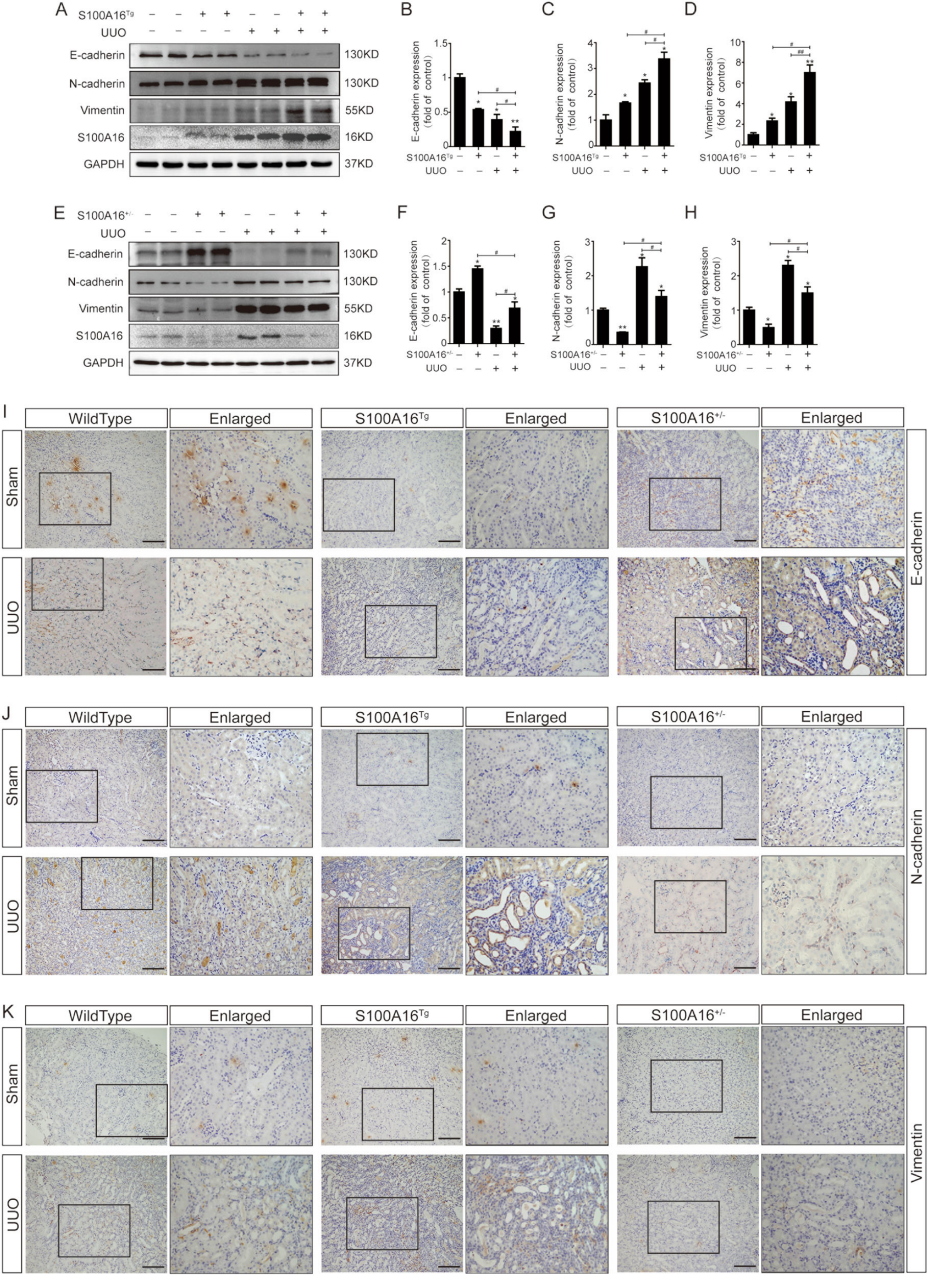
S100A16 promotes EMT in the fibrotic kidney. **a**–**d** Representative immunoblotting and corresponding semi-quantification of E-cadherin, N-cadherin, vimentin, and S100A16 protein expression in obstructed kidneys of wild type and S100A16^Tg^ mice. *p < 0.05, **p < 0.01 vs. wild type sham groups; ^#^*p* < 0.05, ^##^*p* < 0.01. **e**–**h**^.^ Representative immunoblotting and corresponding semi-quantification of E-cadherin, N-cadherin, vimentin, and S100A16 protein expression in obstructed kidneys of wild type and S100A16^+/−^ mice. *p < 0.05, **p < 0.01 vs. wild type sham grou*p*s; ^#^*p* < 0.05, ^##^*p* < 0.01. **i**–**k** Immunohistochemical staining of E cadherin^,^ N-cadherin, vimentin in the obstructed kidneys of WT, S100A16^Tg^, and S100A16^+/−^ mice. Scale bar = 50 μm.

Comparing to the wild-type control animals, in S100A16^Tg^ mouse kidneys the E-cadherin expression was lower, and further decreased at day 7 after UUO (Fig. 4a); whereas; the specific markers for mesenchymal cells (N-cadherin and vimentin) were higher, and their expressions were increased further at 7 days after UUO. Quantitative data for the expressions of E-cadherin, N-cadherin, and vimentin are shown in Fig. 4b–d. Knockout of S100A16 in mice (S100A16^+/−^) led to a reduction in N-cadherin and vimentin expression, accompanied with an increase in E-cadherin expression (Fig. 4e). Quantitative data for E-cadherin, N-cadherin, and vimentin in S100A16^+/−^ mice kidneys are presented in Fig. 4f–h. To confirm the expression of EMT markers, we performed immunohistochemistry staining using renal tissues in WT, S100A16^Tg^ and S100A16^+/−^ mice before and after UUO. The results were consistent with the biochemical data described above (Fig. 4i–k). These results demonstrated that S100A16 promoted the EMT process of fibrotic kidney cells in vivo.

### S100A16 physically interacts with Myosin-9

We next used liquid chromatography and tandem mass spectrometry (LC-MS/MS) to screen the particular S100A16 binding proteins in HK-2 cells stably expressing S100A16. The cell lysates were immunoprecipitated with an anti-S100A16 antibody. As shown in Table 1, the peptides identified by LC-MS/MS were categorized based on their cell metabolic functions, which included trypsinogens, calcium-binding proteins, calcium channel proteins, cell skeleton proteins, protein translocation factors, heat shock proteins, transcriptional regulators, neurodevelopment factors, and enzymes. Among top candidate S100A16 binding proteins, myosin-9 (Myh9) was one of the top five cell skeleton proteins that were functionally related to the EMT.

**Table 1:** Analysis of protein interaction about S100A16 in MS.

Function	Gene	Protein	Scramble TSC^a^	S100A16^OE^ TSC^b^	Value
Trypsinogens	PRSS1	Protease serine 1 OS = Homo sapiens GN = PRSS1 PE = 1 SV = 1	+	+	7
	PRSS3	Isoform 5 of Trypsin-3 OS = Homo sapiens GN = PRSS3	+	+	3
	MGAM	Maltase-glucoamylase intestinal OS = Homo sapiens GN = MGAM PE = 1 SV = 2	+	−	2
	PRSS3P2	Putative trypsin-6 OS = Homo sapiens GN = PRSS3P2 PE = 5 SV = 2	+	−	1
Calcium-binding proteins and calcium channel	S100A16	Protein S100-A16 OS = Homo sapiens GN = S100A16 PE = 1 SV = 1 user pasted sequence	+	+	7
	CALM1	Calmodulin OS = Homo sapiens GN = CALM1 PE = 1 SV = 2	+	+	6
	RYR2	Ryanodine receptor 2 OS = Homo sapiens GN = RYR2 PE = 1 SV = 2	−	+	3
	RYR1	Isoform 2 of Ryanodine receptor 1 OS = Homo sapiens GN = RYR1	−	+	1
	EFHD2	EF-hand domain-containing protein D2 OS = Homo sapiens GN = EFHD2 PE = 1 SV = 1	−	+	1
	MRVI1	Isoform 4 of Protein MRVI1 OS = Homo sapiens GN = MRVI1	−	+	2
	ITPR1	Isoform 4 of Inositol 1 4 5-trisphosphate receptor type 1 OS = Homo sapiens GN = ITPR1	+	−	1
Skeleton proteins	VIM	Vimentin OS = Homo sapiens GN = VIM PE = 1 SV = 4	+	+	9
	MYH9	Myosin-9 OS = Homo sapiens GN = MYH9 PE = 1 SV = 4	−	+	9
	ACTB	Actin cytoplasmic 1 OS = Homo sapiens GN = ACTB PE = 1 SV = 1	−	+	7
	MYL6	Myosin light polypeptide 6 OS = Homo sapiens GN = MYL6 PE = 1 SV = 1	−	+	3
	TPM3	Isoform 2 of Tropomyosin alpha-3 chain OS = Homo sapiens GN = TPM3	+	+	3
	TPM4	Tropomyosin alpha-4 chain OS = Homo sapiens GN = TPM4 PE = 1 SV = 3	+	–	2
	TTBK2	Tau-tubulin kinase 2 OS = Homo sapiens GN = TTBK2 PE = 1 SV = 2	+	−	1
	KIAA0368	Proteasome-associated protein ECM29 homolog OS = Homo sapiens GN = KIAA0368 PE = 1 SV = 1	+	−	1
	MYL10	Myosin regulatory light chain 10 OS = Homo sapiens GN = MYL10 PE = 2 SV = 2	−	+	1
	MAP4	Microtubule-associated protein OS = Homo sapiens GN = MAP4 PE = 1 SV = 1	−	+	1
	TPM1	Isoform 9 of Tropomyosin alpha-1 chain OS = Homo sapiens GN = TPM1	−	+	1
	TPM2	Isoform 2 of Tropomyosin beta chain OS = Homo sapiens GN = TPM2	+	−	1
	ECM1	Isoform 2 of Extracellular matrix protein 1 OS = Homo sapiens GN = ECM1	−	+	1
	ENC1	Isoform 2 of Ectoderm-neural cortex protein 1 OS = Homo sapiens GN = ENC1	−	+	1
	COL6A5	Isoform 2 of Collagen alpha-5(VI) chain OS = Homo sapiens GN = COL6A5	+	−	1
Protein translocation	GOLGA4	Golgin subfamily A member 4 (Fragment) OS = Homo sapiens GN = GOLGA4 PE = 1 SV = 1	+	+	2
	PLIN3	Perilipin-3 OS = Homo sapiens GN = PLIN3 PE = 1 SV = 3	−	+	1
	KIF26B	Isoform 2 of Kinesin-like protein KIF26B OS = Homo sapiens GN = KIF26B	−	+	1
	KIF1B	Isoform 2 of Kinesin-like protein KIF1B OS = Homo sapiens GN = KIF1B	−	+	1
	EXOC7	Isoform 2 of Exocyst complex component 7OS = Homo sapiens GN = EXOC7	+	+	1
	ABCA2	ATP-binding cassette sub-family A member 2 OS = Homo sapiens GN = ABCA2 PE = 1 SV = 3	−	+	1
Heat shock protein	HSPA5	78 kDa glucose-regulated protein OS = Homo sapiens GN = HSPA5 PE = 1 SV = 2	+	+	10
Transcriptional regulation	NIPBL	Nipped-B-like protein OS = Homo sapiens GN = NIPBL PE = 1 SV = 2	+	+	9
	YBX1	Nuclease-sensitive element-binding protein 1 OS = Homo sapiens GN = YBX1 PE = 1 SV = 3	−	+	9
	TACC2	Transforming acidic coiled-coil-containing protein 2 OS = Homo sapiens GN = TACC2 PE = 1 SV = 1	−	+	7
	RPRD2	Regulation of nuclear pre-mRNA domain-containing protein 2 OS = Homo sapiens GN = RPRD2 PE = 1 SV = 1	−	+	3
	EEF1A1P5	Putative elongation factor 1-alpha-like 3 OS = Homo sapiens GN = EEF1A1P5 PE = 5 SV = 1	+	+	3
	MSH5	MutS protein homolog 5 OS = Homo sapiensGN = MSH5 PE = 1 SV = 1	+	−	2
	ZNF462	Isoform 3 of Zinc finger protein 462 OS = Homo sapiens GN = ZNF462	+	−	1
	SETD1B	Histone-lysine N-methyltransferase SETD1B OS = Homo sapiens GN = SETD1B PE = 1 SV = 3	+	−	1
	HIST1H1C	Histone H1.2 OS = Homo sapiens GN = HIST1H1C PE = 1 SV = 2	−	+	1
	FANCB	Fanconi anemia group B protein (Fragment) OS = Homo sapiens GN = FANCB PE = 1 SV = 1	−	+	1
	ETAA1	Ewing’s tumor-associated antigen 1 OS = Homo sapiens GN = ETAA1 PE = 1 SV = 2	−	+	1
	ZFHX4	Zinc finger homeobox protein 4 OS = Homo sapiens GN = ZFHX4 PE = 1 SV = 1	+	−	1
	DDX60L	Probable ATP-dependent RNA helicase DDX60-like OS = Homo sapiens GN = DDX60L PE = 2 SV = 2	−	+	1
Neurodevelopment	USH2A	Usherin OS = Homo sapiens GN = USH2A PE = 1 SV = 3	−	+	2
	NAV3	Isoform 3 of Neuron navigator 3 OS = Homo sapiens GN = NAV3	+	−	2
	PLXNA2	Plexin-A2 OS = Homo sapiens GN = PLXNA2 PE = 1 SV = 4	−	+	1
	ASPM	Abnormal spindle-like microcephaly-associated protein OS = Homo sapiens GN = ASPM PE = 1 SV = 2	−	+	1
Enzyme	ATP5B	ATP synthase subunit beta mitochondrial OS = Homo sapiens GN = ATP5B PE = 1 SV = 3	−	+	2
	PRSS37	Probable inactive serine protease 37 OS = Homo sapiens GN = PRSS37 PE = 2 SV = 1	−	+	1
	PLPP6	Phospholipid phosphatase 6 OS = Homo sapiens GN = PLPP6 PE = 1 SV = 3	−	+	1
	ATL2	Isoform 5 of Atlastin-2 OS = Homo sapiens GN = ATL2	+	−	1
	AKAP6	A-kinase anchor protein 6 OS = Homo sapiens GN = AKAP6 PE = 1 SV = 3	+	−	1

Scramble HK2 cells transfected with scramble lentivirus.

S100A16^OE^ HK2 cells transfected with S100A16 lentivirus.

*TSC* total spectral counts.

**Fig. 5:**
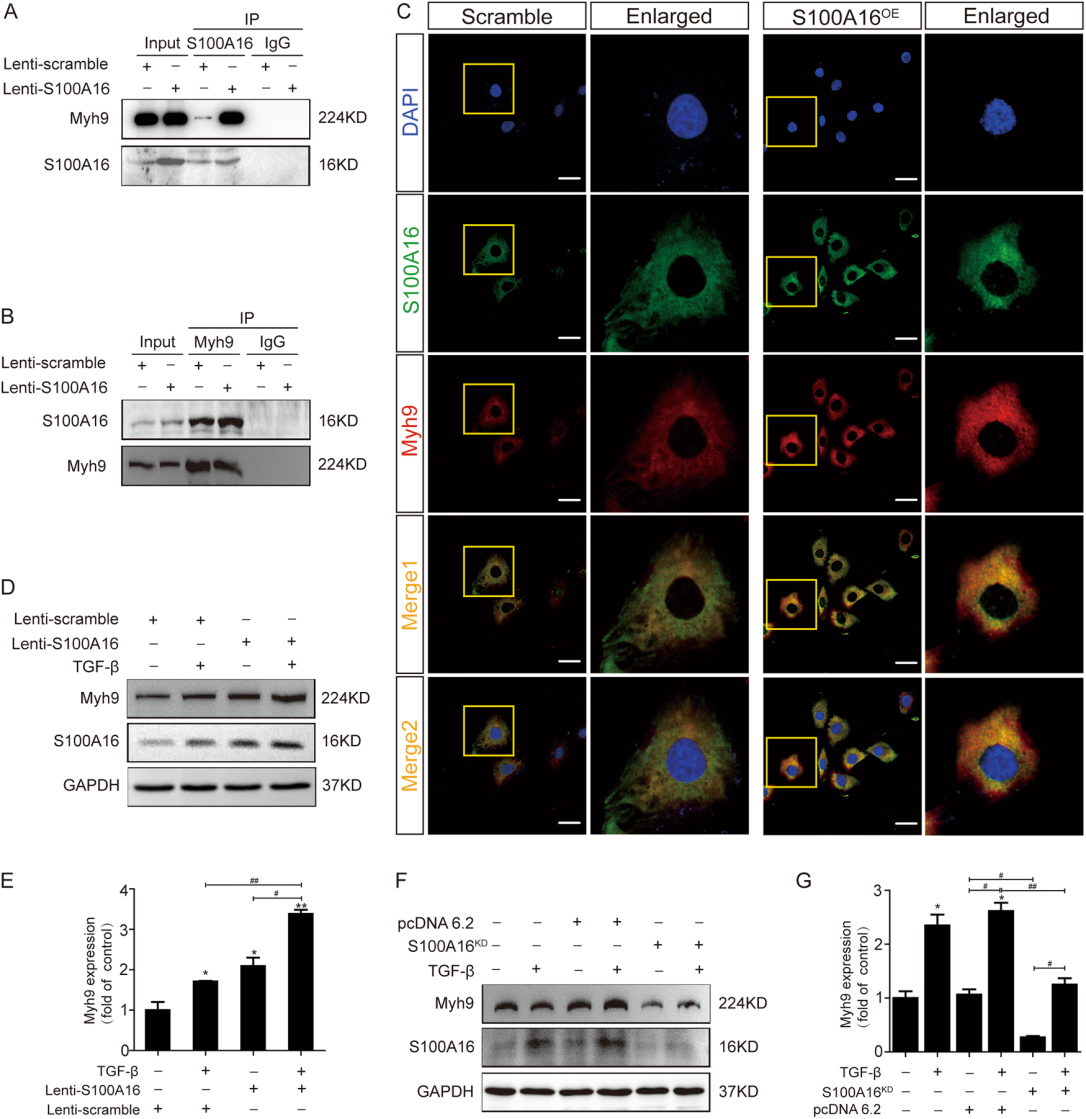
Myh9 physically interacts with S100A16. **a**, **b** An interaction between Myh9 and S100A16 was detected in the co-immunoprecipitation analysis in normal and lenti-S100A16 virus-treated HK-2 cells. The binding between S100A16 and Myh9 was confirmed in immunoprecipitation assays performed using anti-Myh9 antibodies and blotted with anti-S100A16 antibodies in lenti-scrambled and lenti-S100A16 virus treated HK-2 cells. **c** S100A16 and Myh9 partially colocalized in normal and S100A16 overexpressing HK-2 cells. Scale bar = 20 μm. **d**–**g** HK-2 cells transfected with lenti-scrambled, lenti-S100A16 virus, and S100A16 knockdown plasmids were stimulated with TGF-β (20 ng/ml). Representative bands of western blots are shown for the expression of Myh9. *p < 0.05, **p < 0.01 vs. control; ^#^*p* < 0.05, ^##^*p* < 0.01.

The binding of S100A16 to Myh9 was confirmed by co-IP using S100A16 antibodies to isolate the protein complex from WT or SA100A16-overexpressing HK-2 cells followed by blotting using Myh9 antibodies; no Myh9 signal was detected when IgG was used as a control. Such physical interaction between S100A16 and Myh9 was detectable under endogenous conditions after S100A16 overexpression (Fig. 5a).

This interaction was also supported by IP experiments using Myh9 antibodies and blotting with S100A16 antibodies (Fig. 5b). Notably, immunofluorescence staining images indicated a marked co-localization of S100A16 and Myh9 (Fig. 5c), in agreement with the biochemical data (Fig. 5a, b). Furthermore, it appears that S100A16 overexpression, similar to the effects by TGF-β treatment, significantly induced Myh9 protein expression in HK-2 cells (Fig. 5d). Quantitative data for Myh9 expression in HK-2 cells treated with TGF-β are presented in Fig. 5e. In HK-2 cells where S100A16 is knocked down, however, showed an opposite pattern with or without TGF-β stimulation (Fig. 5f, g). Vimentin (a cytoskeleton protein) and GRP78 were identified by LC-MS/MS (Table 1) and were also confirmed to bind with S100A16 in HK-2 cells using co-IP techniques (Supplemental Fig. 2).

### Myosin-9 is required for S100A16-induced EMT in HK-2 cells

**Fig. 6:**
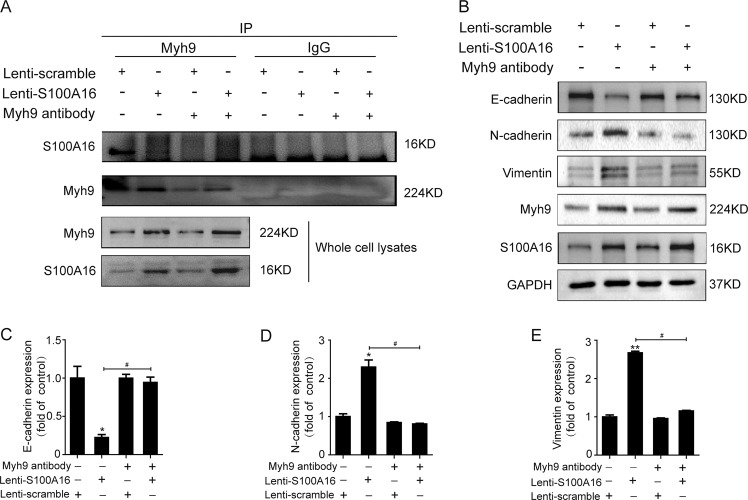
Myosin-9 is required for the S100A16-induced EMT in HK-2 cells. **a** Pre-transfection of normal and S100A16-overexpressing (S100A16^OE^) HK-2 cells with the antibody against Myh9 reduced the levels of binding between S100A16 and Myh9. **b**–**e** Representative bands of western blots are shown for the expression of Myh9, E-cadherin, N-cadherin, vimentin, and S100A16 in normal and S100A16 overexpressing HK-2 cells after inhibition of Myh9 by antibody transfection. *p < 0.05, **p < 0.01 vs. scrambles; ^#^*p* < 0.05.

The binding between S100A16 and Myh9 was further confirmed by transfecting the antibody against Myh9 into HK-2 cells by Pro-Ject^TM^ protein transfection as a way to compete with the binding with S100A16. As shown in Fig. 6a, S100A16 overexpression augmented the interaction with Myh9. However, this association between S100A16 and Myh9 was attenuated by pre-transfection of the HK-2 cells with the Myh9 antibody.

The role of Myh9 in the process of EMT induction by S100A16 was also evaluated by competition experiments conducted in HK-2 cells using a Myh9-targeting antibody. As shown in Fig. 6b, S100A16 overexpression decreased E-cadherin expression and induced N-cadherin and vimentin expression in HK-2 cells, as part of the EMT pathogenetic process, in agreement with the data shown in Fig. 3a. However, these reciprocal expression changes in the EMT markers were suppressed when the cells were pre-transfected with the antibody against Myh9. Quantitative data for EMT marker expression in HK-2 cells treated with or without Myh9 antibody are presented in Fig. 6c–e. Those findings suggested that Myh9 was required for the S100A16-induced promotion of the EMT in renal tubular injury.

### Increased S100A16 expression drives Ca^2+^ accumulation in the cytoplasm and promotes cytoskeleton reorganization in HK-2 cells

**Fig. 7:**
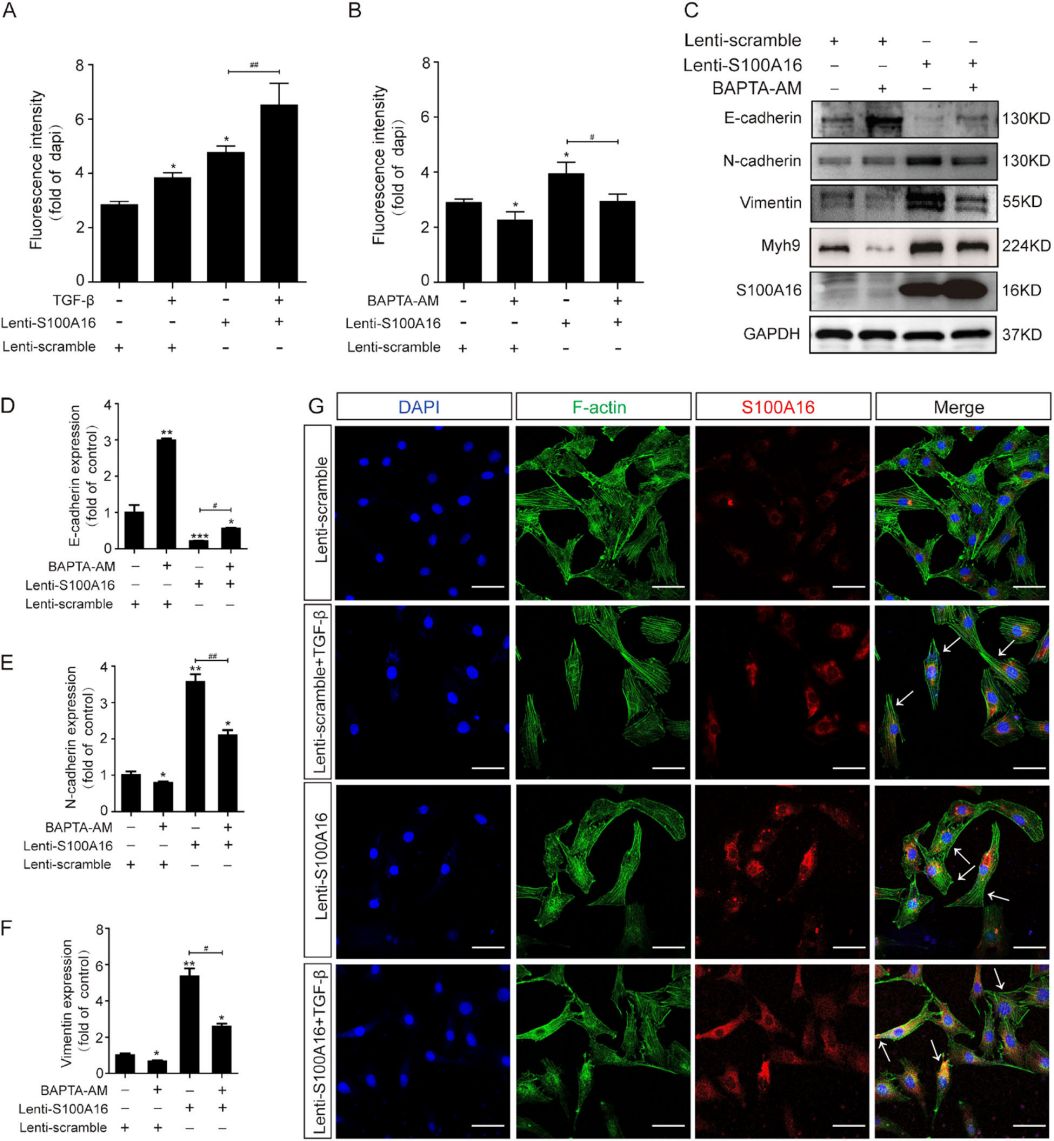
Increased S100A16 expression in HK-2 cells drives Ca^2+^ accumulation in the cytoplasm and promotes cytoskeleton reorganization. **a**, **b** Normal and S100A16-overexpressing HK-2 cells were treated with TGF-β (20 ng/ml) or BAPTA-AM (10 µM), and the intracellular calcium concentration was measured using Rhod-2 AM, a fluorescent Ca^2+^ indicator probe. **c**–**f**. Representative bands of western blots are shown for the expression of Myh9, E-cadherin, N-cadherin, vimentin, and S100A16 in normal and S100A16-overexpressing HK-2 cells after BAPTA-AM treatment. *p < 0.05, **p < 0.01 vs. scrambled; ^#^*p* < 0.05, ^##^*p* < 0.01. **g** Representative images showing the reorganization of F-actin protein in S100A16-overexpressing HK-2 cells after treatment with TGF-β (20 ng/ml) (arrows, cytoskeleton remodeling HK-2 cells). Scale bar = 20 μm.

S100A16 is a calcium-binding signaling protein. We used the fluorescent probe (Rhod-2 AM) loading assays to determine the intracellular calcium concentrations in S100A16-overexpressing HK-2 cells. As shown in Fig. 7a, the Ca^2+^ fluorescence signal was significantly increased in HK-2 cells overexpressing S100A16, indicating that higher S100A16 led to more Ca^2+^ release into the cytoplasm. Consistently, a pretreatment with the Ca^2+^ chelator BAPTA-AM (10 µM) markedly decreased the intracellular calcium concentration in the cells (Fig. 7b). Fluorescence images were shown in supplemental Fig. 3. BAPTA-AM treatment also suppressed the induction of the EMT evidenced by increased E-cadherin expression and decreased N-cadherin and vimentin expression in the S100A16-overexpressing HK-2 cells (Fig. 7c). Quantitative data for EMT marker expressions in HK-2 cells treated with BAPTA-AM are presented in Fig. 7d–f. These results indicate that cytoplasmic Ca^2+^ release may mediate the S100A16 induced EMT.

The morphological changes in S100A16-overexpressing HK-2 cells were examined by immunofluorescence microscopy. Under the basal condition, F-actin staining revealed an organized network of thin, short, noncontractile actin filaments in the cytoplasm (Fig. 7g); whereas S100A16 overexpression induced a reorganization of the F-actin into thicker and more bundled stress fibers, as did the treatment with TGF-β (Fig. 7g). Such morphology change is consistent with the transition of an epithelial cell form to that of spindle-like mesenchymal cells.

## Discussion

**Fig. 8:**
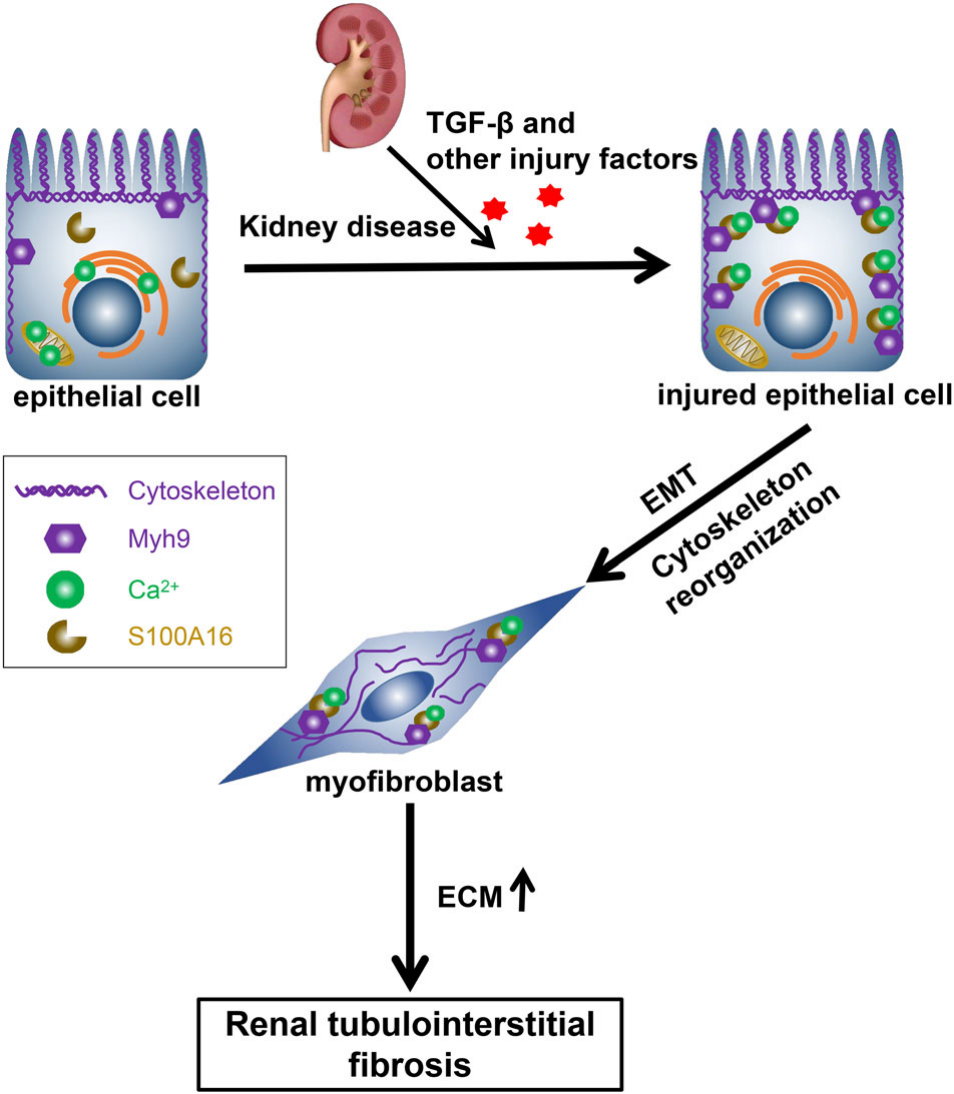
Schematic diagram showing the EMT and cytoskeleton reorganization induced by the combined actions of S100A16, Myh9, and Ca^2+^. Kidney disease, TGF-β, and other injury factors result in combined actions of S100A16, Myh9, and Ca^2+^ in injured epithelial cells. The EMT and cytoskeleton reorganization lead to the transition of renal epithelial cells into myofibroblasts, followed by accumulation of extracellular matrix and aggravation of renal tubulointerstitial fibrosis.

The EMT is often accompanied by a switch in the expression of E-cadherin and N-cadherin^4,21^. During the pathological processes associated with the EMT, the expression of epithelial genes that maintain epithelial cell-cell adhesions is repressed, and the expression of mesenchymal genes promoting cell migration and invasion is activated. In this study, S100A16 expression induced the downregulation of E-cadherin (an adherent junction protein) and the upregulation of N-cadherin (a mesenchymal cell–cell adhesion protein), vimentin (an intermediate filament protein), and fibronectin and collagen (extracellular matrix proteins). S100A16 knockout substantially attenuated both the TGF-β-induced EMT in HK-2 cells and the kidney injury associated with the UUO mouse model. Therefore, S100A16 participated in the TGF-β-induced fibrogenic process in tubular epithelial cells and promoted the fibrotic kidney condition associated with the EMT. S100A16 expression also increased the intracellular calcium concentration and interacted with Myh9 to promote cytoskeleton reorganization that led to the changes in cell shape associated with the EMT. These findings confirmed the contribution of S100A16 to renal tubulointerstitial fibrosis (Fig. 8).

The gene MYH9, encoding heavy chain of non-muscle myosin class II, isoform A (NM IIA), participates in a variety of biological processes inside of cells including translocation of the actin cytoskeleton. The functions of NM IIA are regulated by phosphorylation of its 20 kDa light chain and Mhy9, and by interactions with other proteins^22^. It is reported that NM IIA localization and filament assembly can be modulated by interaction with S100A4, a member of the S100 family^7,23,24^. In our study, we found that S100A16 as a new member of S100 family was a binding protein of Myh9 in HK-2 cells, which contribute to the candidate group of NM IIA interaction proteins, and further to modulate a number of cytoskeleton translocation.

MYH9 missense mutations have been shown to cause an autosomal-dominant disorder, termed MYH9-related disease (MYH9-RD), and associated with other human diseases such as chronic kidney disease, non-syndromic deafness, and cancer^22,25^. Genetic variations in Myh9 are associated with a predisposition to chronic kidney disease (CKD) and other renal injury including human immunodeficiency virus-associated collapsing glomerulopathy, focal segmental glomerulosclerosis, hypertension-attributed end-stage kidney disease, and diabetes-attributed end stage kidney disease. Moreover, the E1 haplotype of Myh9 has been linked to an increased prevalence of glomerulosclerosis^26^ and non-diabetic end stage renal disease^27^ in African Americans and in Hispanic Americans^25,28^.

It is reported that the MYH9 kidney risk variant is characterized by multiple intronic single nucleotide polymorphisms (SNPs), but the causative variant has not been identified. In addition, there is expression of MYH9 in podocytes, which links glomerular pathophysiology^25^. Our study here demonstrated that MYH9 expressed in renal tubular epithelial cells and associated with renal tubulointerstitial injury.

The biological function of Myh9 normally depends on the calcium concentration, and S100A16 is a calcium binding protein. In our study, we observed that blocking calcium through pretreatment with BAPTA-AM will attenuate the effects of S100A16 on EMT markers in HK-2 cells, which confirmed that cytoplasmic Ca^2+^ release mediate S100A16-induced EMT. The results demonstrated that Myh9 was a specific target that binds with S100A16 to play a role in cytokinesis, cell shape, and cytoskeleton reorganization. However, we don’t know the reason why Myh9 protein level was decreased with pretreatment of BAPTA-AM, which need further investigation in future.

One important event in EMT is the remodeling of actin filaments. The reorganization of F-actin corresponds with the cell morphology changes occurring during the EMT^5,6^. The cortical thin bundles of the actin filaments in epithelial cells undergo remodeling into thick contractile stress fibers in transdifferentiated mesenchymal cells by a mechanism involving RhoA^4^. In TGF-β-induced EMT, RhoA is required for localization of E-cadherin at cell–cell adhesions and to promote the transition to a mesenchymal cell morphology^29,30^. Genome-wide expression studies have shown that genes encoding the actin cytoskeleton are consistently upregulated in TGF-β-induced EMT cell models^31,32^. The moesin protein, which is part of the ezrin/radixin/moesin (ERM) transcriptional pathway, has been shown to promote EMT by regulating adhesion and contractile elements in efficient actin filament remodeling^33,34^. Knockdown of moesin expression by shRNA induced thinner and less stable actin bundles, an incomplete morphological change, and a decreased invasive capacity of the cells^15,35^. These studies indicated that transcriptional regulation drives the EMT-mediated progressive remodeling of actin filament architectures. However, the biological and functional significance of this regulated expression, and whether the EMT requires reorganization of the actin cytoskeleton by regulatory proteins other than RhoA and moesin, remain to be established.

Taken together, the findings of the present study support an involvement of S100A16 in the EMT process through its binding with Myh9. The subsequent alteration of F-actin remodeling then leads to renal tubulointerstitial fibrosis. However, the mechanism by which the EMT regulates S100A16 expression needs further investigation.

## Supplementary information

Suppl table 1

suppl Figure legend

Suppl Figure 1

Suppl Figure 2

Suppl Figure 3
