# Supplementary material for: Interaction of calcium binding protein S100A16 with myosin-9 promotes cytoskeleton reorganization in renal tubulointerstitial fibrosis
Source: Cell Death Dis. 2020 Feb 24;11(2):146. doi: 10.1038/s41419-020-2337-z (PMC7039973; doi:10.1038/s41419-020-2337-z)
Supplement: Supplementary file 1 — Suppl table 1 [file 41419_2020_2337_MOESM1_ESM.docx]

|  | WT (n=16) | | *P* value | S100A16^Tg^ (n=16) | | *P* value | S100A16^+/-^ (n=16) | | *P* value |
| --- | --- | --- | --- | --- | --- | --- | --- | --- | --- |
|  | Sham (n=8) | UUO (n=8) |  | Sham (n=8) | UUO (n=8) |  | Sham (n=8) | UUO (n=8) |  |
| Scr (μmol/L) | 22.7±1.58 | 25.75±0.89 | 0.0004 | 26.35±1.52 | 31.00±3.12 | 0.0020 | 19.75±2.80 | 22.00±2.39 | 0.1061 |
| BUN (mmol/L) | 9.31±0.89 | 11.06±1.03 | 0.0044 | 10.99±0.53 | 13.20±0.97 | 0.0001 | 7.69±0.46 | 9.34±0.83 | 0.0004 |

**Supplemental Table 1. Renal functional parameters in mice**
