# Supplementary material for: Interaction of calcium binding protein S100A16 with myosin-9 promotes cytoskeleton reorganization in renal tubulointerstitial fibrosis
Source: Cell Death Dis. 2020 Feb 24;11(2):146. doi: 10.1038/s41419-020-2337-z (PMC7039973; doi:10.1038/s41419-020-2337-z)
Supplement: Supplementary file 2 — suppl Figure legend [file 41419_2020_2337_MOESM2_ESM.docx]

**Supplemental Figure Legend**

**Supplemental Figure 1. The efficacy of overexpress and knockdown S100A16 was evaluated by real-time PCR.** Semi-quantitative analysis of S100A16 mRNA expression levels after S100A16-overexpressing (A) or knockdown (B). ****p*<0.001

**Supplemental Figure 2. S100A16 physically interacts with vimentin.** The cell lysates were from HK-2 cells transfected with scramble or S100A16 lentivirus. Positive signal was detected by western blot with S100A16 antibodies in immunoprecipitation experiments performed using lysates from HK-2 cells transfected with scramble or lenti-S100A16, but IPs performed with an anti-IgG as a control yielded no vimentin or GRP78 signal.

**Supplemental Figure 3. Increased S100A16 expression in HK-2 cells drives Ca^2+^ accumulation in the cytoplasm.** Representative images showing the Ca^2+^ accumulation in HK-2 cells infected with lenti-S100A16 after treatment with TGF-β or BAPTA-AM (a Ca^2+^ chelator). Scale bar = 50 μm. The intracellular calcium concentration was measured using Rhod-2 AM.
