# Supplementary figures and images for: Interaction of calcium binding protein S100A16 with myosin-9 promotes cytoskeleton reorganization in renal tubulointerstitial fibrosis
Source: Cell Death Dis. 2020 Feb 24;11(2):146. doi: 10.1038/s41419-020-2337-z (PMC7039973; doi:10.1038/s41419-020-2337-z)

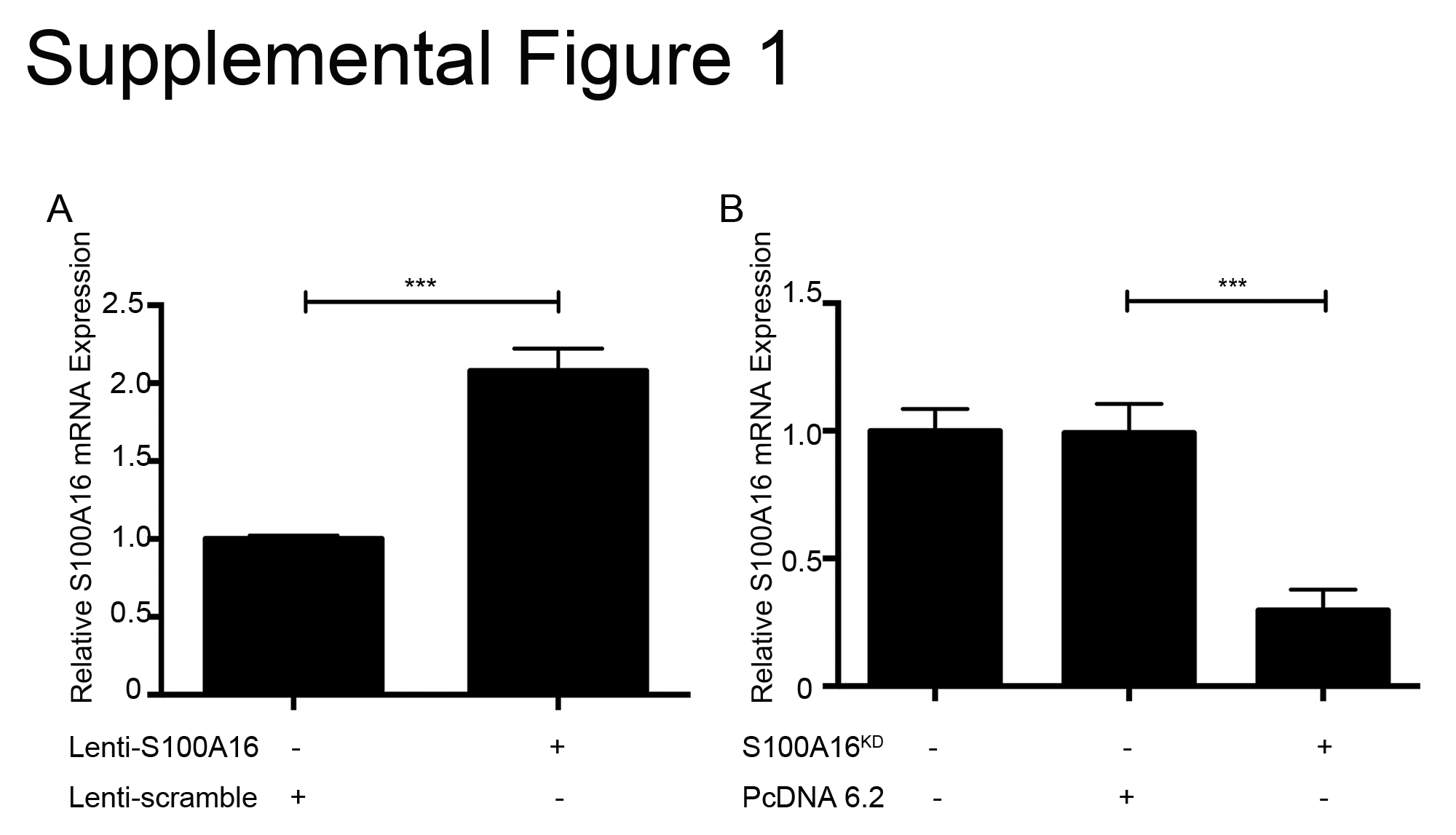

Supplement: Supplementary file 3 — Suppl Figure 1 [file 41419_2020_2337_MOESM3_ESM.tif]

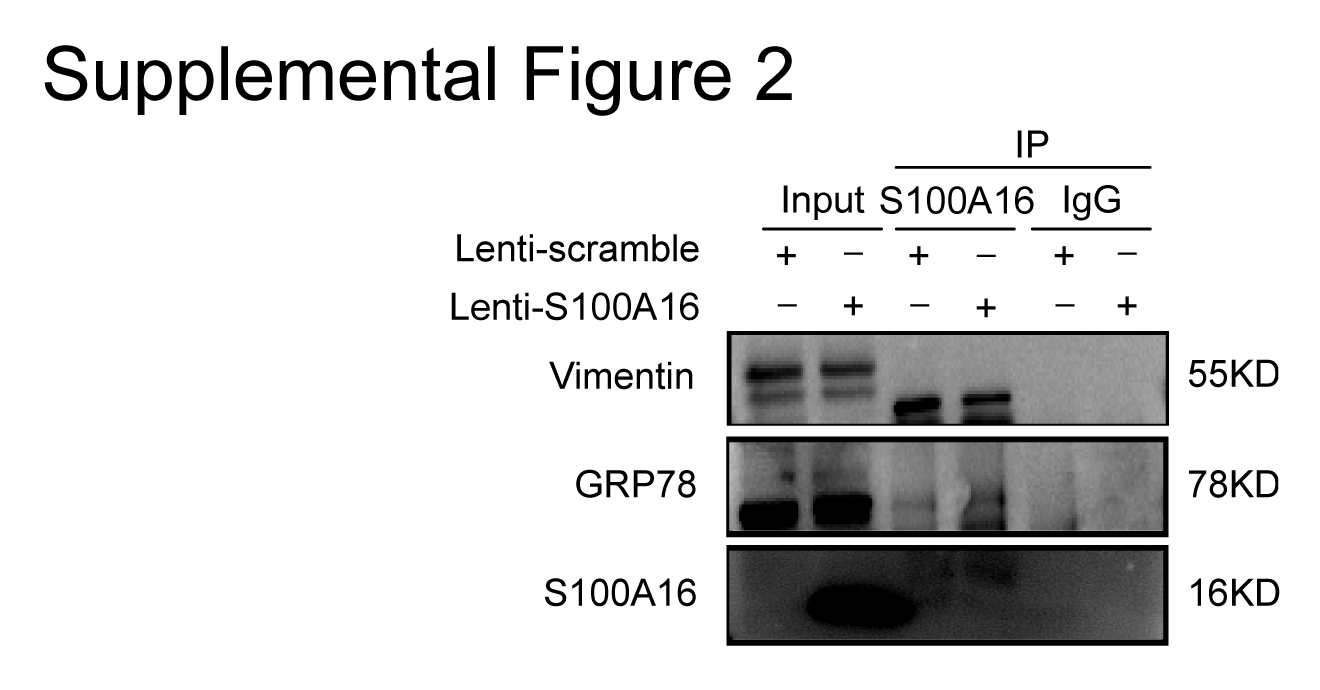

Supplement: Supplementary file 4 — Suppl Figure 2 [file 41419_2020_2337_MOESM4_ESM.tif]

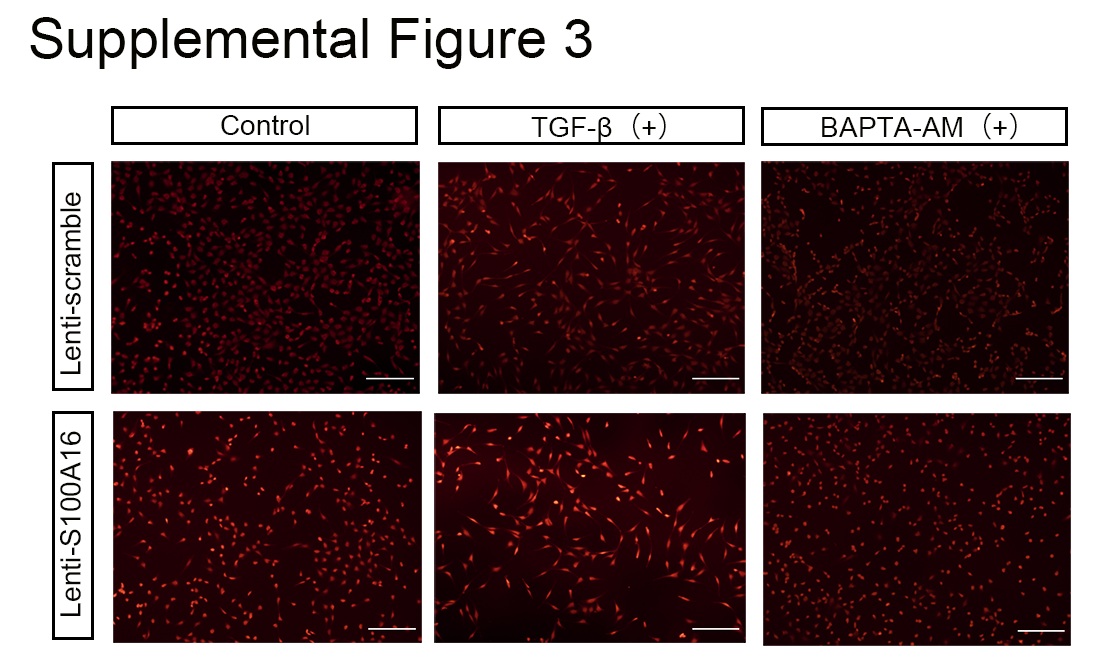

Supplement: Supplementary file 5 — Suppl Figure 3 [file 41419_2020_2337_MOESM5_ESM.tif]
